# Synergistic effects in bimetallic Pd–CoO electrocatalytic thin films for oxygen evolution reaction

**DOI:** 10.1038/s41598-020-71389-w

**Published:** 2020-09-02

**Authors:** Muhammad Ali Ehsan, Abbas Saeed Hakeem, Abdul Rehman

**Affiliations:** 1 Center of Research Excellence in Nanotechnology (CENT), King Fahd University of Petroleum & Minerals, Dhahran, 31261 Saudi Arabia; 2 Department of Chemistry, King Fahd University of Petroleum and Minerals, Dhahran, 31261 Saudi Arabia

**Keywords:** Electrocatalysis, Catalyst synthesis

## Abstract

Bimetallic catalysts due to the synergistic effects often outperform their single-component counterparts while exhibiting structure and composition-dependent enhancement in active sites, thereby having the potential to improve the current density and over-potential parameters in the water oxidation reaction. Herein, we demonstrate a simple and rapid, yet highly efficient method to fabricate Pd–CoO films of immaculate homogeneity as characterized using different imaging and spectroscopic techniques. The SEM images revealed that the films were composed of bimetallic spherical granules wherein both metals were uniformly distributed in an atomic ratio of ~ 1:1. The time-dependent investigations of the film fabrication behavior demonstrated that the films formed in shorter deposition times (1–2 h) display more porous character, allowing better access to the reaction centers. This character was transcribed into their enhanced electrocatalytic performance toward the oxygen evolution reaction (OER). Using this specific bimetallic formulation, we could attain a low over-potential of 274 mV for a current density of 10 mA cm^−2^, whereas the high current density value of > 200 mA cm^−2^ was achieved while still under 600 mV of over-potential. The cycling and current generation stability was also found to be sufficiently high, which can only be attributed to the facile electron transfer processes and a higher number of active sites available in homogeneous bimetallic films.

## Introduction

Electrocatalytic water oxidation is an extensively studied reaction due to its importance in industrial water splitting for hydrogen-based economy^1^. Such a non-reliance on fossil fuels can enforce a change towards a more sustainable society with a lesser burden of pollution on the climate. However, this anodic reaction is a limiting factor in the large scale electrolysis of water^2^. The process includes four proton-coupled electron transfers and two oxygen atoms bonding together, making it electrochemically sluggish because of high energy demands^3^. The result is the high over-potentials, and low current densities, whereas the target is to achieve an industrial standard current density of more than 100 mA cm^−2^ with the least over-potential possible^4^. Thus, the materials with the ability to catalyze this reaction are of high significance not only for water splitting based hydrogen generation^5,6^, but also for oxygen evolution reactions (OER) in metal-air batteries^7,8^, or other oxidation reactions such as the oxidation of CO^9^ for the purification of hydrogen fuel obtained via currently accustomed fossil fuels based production. While the oxides of Iridium and Ruthenium still remains the benchmark in catalyzing this process^10^, the search for the earth-abundant low-cost substitute continues^5,11^. An illustrative outcome of this search is the oxides of transition metals (e.g., Ni, Co, Fe) showing structures and compositions similar to the active center of spinels or other oxygen-evolving complexes^12–14^. From these materials, the oxides of cobalt, especially the CoO, have shown promising results both in terms of overpotential and current density^15–17^. However, the electrocatalytic activity of such materials is often impaired by the poor conductivity on offer. Moreover, these materials are prone to durability and stability issues, which are the result of reconstructional, segregative, and/or redox changes occurring in the activation process or during the reaction itself^18,19^.

A newly proposed strategy to overcome these issues of conductivity and stability in heterogeneous cobalt catalysts is to build synergic interfaces^20–22^ between its oxides and the highly active metals such as Pt, Au, or Pd^23^. For instance; it has been recently shown that the Co (IV) entities^24^ in various cobalt oxides have a crucial role as intermediate states, accelerating the oxygen generation at the interphase. The inclusion of more active metals increases the population of Co (IV) on the catalyst and increases the number of active centers, thereby improving the catalytic efficiency^20–23^. Various different protocols are available to create that synergic interface, the simplest of which is to deposit different cobalt oxide films over metal substrates^22^. However, this method requires the bulk of precious metals as the support, which also lacks the desired porosity and contact points to define the real synergy. Our group has also developed cobalt oxide films using this strategy but not over the bulk metal, rather over a 50 nm thick layer of metal evaporated gold^25^, with better synergic effects. Loading the precious metal nanoparticles into cobalt oxide nanostructures and films is another approach for obtaining the composites. Lu et al.^26^, for example, has embedded the Au nanoparticles into the mesoporous cobalt oxide, enhancing the electrochemical performance of the material. However, the mixing of the Au and Co precursors in such cases can lead to the non-homogenous distribution of active sites, barring the electrochemical activity. Qu et al.^23^ has also fabricated mesoporous cobalt oxide embedded with Pt, Pd, and Au nanoparticles with differing mass loadings and high surface area; however, the TEM images show the nanoparticles at distinctive points. As a result, the authors could be able to obtain low onset potentials, yet the current density can rarely reach more than 10 mA cm^−2^. The most appealing approach in this regard is either to alloy the earth-abundant metals with the precious metals as in the case of FePt^27^, NiPd^19^, and CuAu^28^, or to chemically deposit both the metal and metal oxide in a single step^29^. This approach can realize the interfacial synergy to occur at the atomic level and over the whole surface, whether the composite is in the form of a nano-dimensional film or any other nanostructure^20^.

In this work, we intended to build this synergy in the pristine CoO, being the most active form of cobalt in water oxidation, by using a facile single-step methodology of aerosol assisted chemical vapor deposition (AACVD). It has been theoretically shown that CoOx/Pd(100) thin films based model systems in an ultra-high vacuum tend to induce novel structures and peculiar chemical attributes especially related to the Pd metal^22^. It has also been demonstrated in the same work that the CoOx combination with the Pd can provide an activity comparable to pure Pd, but without the poisoning of Pd-metal due to the presence of oxide content. The argument that describes the enhanced activity of such systems is the presence of combined actions of electron tunneling and electron hybridization from Pd, promoted by the thin insulating and semiconducting layer of cobalt oxide. This phenomenon was further supported by the evidence that the segregated Co at the surface changes compositions while exposed to various oxidants, whereas Pd is also driven to migrate to the surface under the changing chemical conditions^20^. Such migration is dependent upon the CoO content and decrease with increasing ratio of CoO. However, the two coexisting species on the surface and in close proximity at the atomic scale can lead to better oxidation kinetics. With this hypothesis, we fabricated the homogenous thin films of Pd–CoO in 1:1 mol ratio under varying time scales of deposition via AACVD. Initially, films were fabricated on plain glass substrates for structural and morphological characterization using SEM/EDX, XRD, and XPS, showing a well-defined, granular structure composed of Pd and Co materials in highly homogenous distribution. This structural and distributional homogeneity is then correlated to the electrochemical performance in catalyzing the oxygen evolution reaction in water splitting. The data demonstrated that the performance of the films could be linked to the growth patterns, whereas the homogeneity of the films leads to the interfacial synergy for efficient water splitting. Thus, this work highlights the benefits of using AACVD in fabricating the films of various metal/metal oxide combinations at ease with exotic morphologies and desired catalytic attributes to be utilized in various applications.

## Material and methods

### Film fabrication procedure

Thin films of bimetallic Pd–CoO were fabricated using aerosol assisted chemical vapor deposition (AACVD) setup as described in our previous works^24,25,29–31^. The chemicals used in this work; Co(III) acetylacetonate Co(acac)_3_, palladium(II) acetylacetonate Pd (acac)_2,_ and toluene were obtained from Sigma Aldrich and were used as received.

Precursor solution for AACVD process was prepared by mixing Co(acac)_3_ (100 mg, 0.280 mmols) and Pd (acac)_2_ (85 mg, 0.280 mmols) in 15 mL of toluene. The solution was stirred for 1 h until both precursors were completely dissolved to produce a transparent green solution. The solvent was evacuated under reduced pressure to obtain the green powder, which was re-dissolved in 20 mL toluene. This transparent homogenous solution was employed in AACVD to fabricate thin films of Pd–CoO for different time periods.

Initially, thin films were made on plain glass substrates, which were cleaned in an ultrasonic bath using different solvents such as doubly distilled water and acetone before being stored in ethanol until the deposition experiments were performed. The substrates were air-dried before being transferred to the AACVD reactor chamber, which was placed in a horizontal tube furnace (CARBOLITE, Model No. 10/25/130) (6″L × 1″D). The reactor tube was heated to the desired deposition temperatures of 475 °C for 10 min to attain the thermal equilibrium.

In each deposition experiment, 20 mL of the solution of dual precursors (Pd & Co) was transferred into a 50 mL round bottom flask, which was immersed in a water bath lying above the piezoelectric modulator of an ultrasonic humidifier (Model No. Cool Mist-plus serial No. ADV-CMP-85956). This piezoelectric humidifier generated an aerosol mist of the solution of precursors. Nitrogen gas (99.9% purity) was used to transfer the aerosol mist into the reaction chamber and over the heated substrate serving as a carrier gas. The exhaust of the reaction was vented into a fume hood. The aerosol delivery was continued for different time periods of 1 h, 2 h, and 3 h with the corresponding samples named as PdCo-1hr, PdCo-2hr, and PdCo-3hr, respectively. Finally, the coated substrates were cooled while still under a continuous flow of nitrogen, and then removed from the chamber once the temperature is below 100 °C.

### Characterization of the films

X-ray diffraction (XRD) patterns were recorded to investigate the structural properties of mixed palladium cobalt oxide thin films, and these experiments were performed using PANanalytical, X'PertHighScore diffractometer at a monochromatic high-intensity Cu*K*_*α*_ (λ = 1.5418 Å) radiation. Field emission gun (FEG)-SEM images were collected to explore the surface morphologies of the films using a Lyra 3 Tescan, microscope at an accelerating voltage of 5 kV, and a working distance of 10 mm. Energy dispersive X-ray (EDX, INCA Energy 200, Oxford Inst.) spectrometer was used to determine atomic ratios of Pd/Co. X-ray photoelectron spectroscopy (XPS) was performed using the ULVAC-PHI Quantera II spectrometer with a 32-channel Spherical Capacitor Energy Analyzer. This experiment was performed under vacuum (1 × 10^–6^ Pa) using monochromatic Al Kα radiation (1,486.8 eV) with a natural energy width of 680 meV. The binding energies were calibrated against carbonaceous C 1*s* line (284.6 eV) reference.

### Electrochemical measurements

All the electrochemical measurements were performed in a three-electrode system where the fabricated PdCo films were used as a working electrode. A spiral Pt wire of thickness 0.25 mm was used as a counter electrode, which was cleaned by immersing into a 20% solution of HNO_3_ for few minutes following washing with MilliQ water before inserting it into the electrochemical setup. Saturated silver-silver chloride (Ag/AgCl in a saturated solution of KCl) was used as the reference electrode. A computer-controlled AUTOLAB potentiostat was employed for these measurements. After the measurements, all the potentials were converted into potentials against the reversible hydrogen electrode (RHE) as per the Nernst equation.

$$E_{RHE} = E_{REF} + E_{0REF} + \, 0.0{59}({\text{p}}H)$$

Before running the experiments, the glassware, as well as the electrochemical cell, were washed by boiling in 1:3 mixture of H_2_SO_4_ and HNO_3_, followed by boiling in water and rinsing with acetone. The drying of this apparatus was done by keeping in an oven at 100 °C for 1 h, as described previously^24^. Cyclic voltammetry and controlled potential bulk electrolysis experiments were all performed in 0.1 M KOH electrolyte solution having the pH 13. Distilled and deionized water was used to make all the solutions for electrochemical studies using the Milli.Q system from Millipore. Linear sweep voltammetry (LSV) was used in order to find the overpotentials and current density profiles of the films during water splitting OER reaction.

## Results and discussion

### Synthesis and characterization of Pd–CoO granular thin films

**Figure 1:**
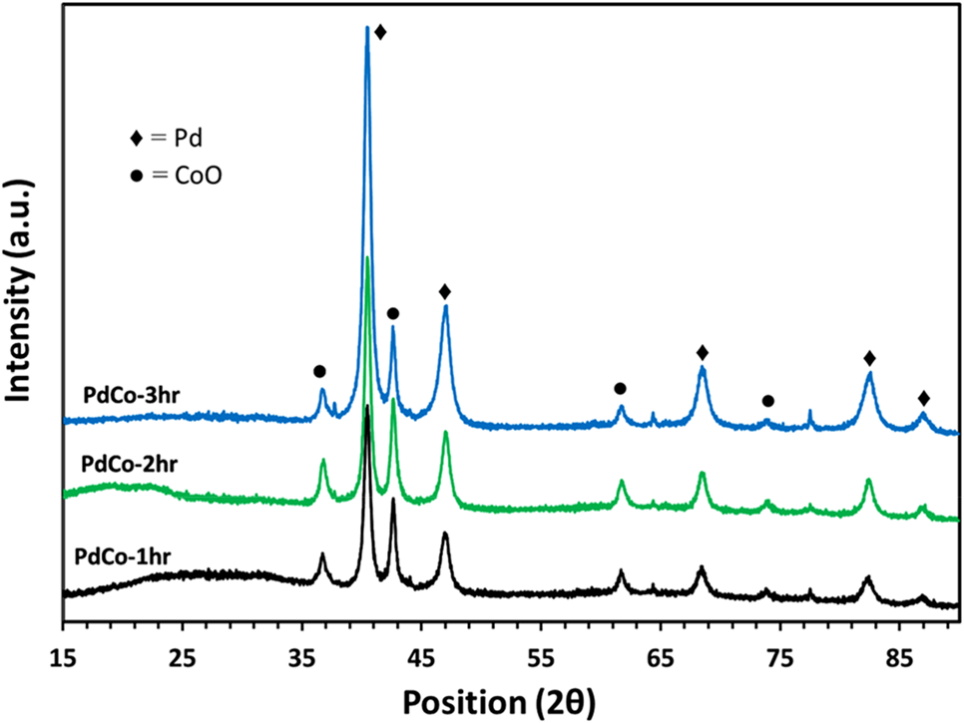
XRD patterns of Pd–CoO composite films deposited on the plain glass substrate at T = 475 °C for different deposition intervals of 1 h (black line), 2 h (green line) 3 h (blue line) via AACVD.

Bimetallic Pd–CoO thin films were synthesized by a facile single-step AACVD procedure, which is based on the generation of aerosol from the precursor solution and depositing the metal/metal oxide components over pre-heated substrates, as shown in our previous work^24,31^. This simple fabrication strategy has been shown to produce exotic nanostructures and nanostructured films in a matter of minutes. The only requirement of this protocol is the availability of a common solvent that can dissolve all the precursors. Toluene was used as the solvent in this study as Pd(acac)_2_ is conveniently soluble in it as compared to polar organic solvents such as methanol. Thus, this method has high superiority over the hydrothermal synthesis^17,32,33^ and hard or soft template-driven fabrication^34^, including the MOFs^21^ or other wet chemistry protocols^35,36^ consuming hours of preparation, purification, and electrode deposition times. It is even more facile than the physical etching or deposition methods^37,38^ as there is no need for sophisticated instrumentation while still having precise control over the composition of the final product. Moreover, different bi and trimetallic thin films with controlled thickness, uniformity of the phases, and strong adhesion to the substrate can be formulated. Here, commercially available precursors, which are very commonly applied in obtaining Pd–Co nanostructures, were used to obtain the films in 1:1 molar ratio of Pd:Co at a relatively low temperature of 475 °C. This ratio was chosen based on a prior understanding that the electro-oxidation performance of Pd–Co materials is heavily dependent upon the composition. During the catalytic reactions, Pd and Co species alternatively migrate to the surface of the nanoparticles or the nano films again depending upon the exposure to the oxidant molecules. This segregation and migration becomes less and less prominent with increasing content of Co as it increases the CoO coverage in the film. The magic number, somehow, as proven experimentally is a little more than 1:1 for Pd:Co. This has been independently shown that Pd–Co combinations show better catalytic performance with at least this much content of Pd, although the Co content can be high in case of Au, or Pt. Therefore, after a number of trials and screening steps, we were able to identify the optimum starting concentrations of precursor solutions that lead to the films having this molar ratio of Pd:Co. Again, multiple different Pd–CoO films were fabricated with the same molar ratio but at different time scales of the deposition, however, three of those films were characterized further based on initial screening, i.e., PdCo-1hr, PdCo-2hr, and PdCo-3hr formed after 1 h, 2 h, and 3 h of deposition. Figure 1 shows the X-ray diffraction patterns (XRD) of Pd–CoO composite thin films fabricated at 475 °C for different time intervals on plain glass substrates.

The XRD patterns of all the composite films revealed the characteristic diffraction peaks of both Pd and CoO crystalline phases, which have been labeled by the signs of filled diamond and filled circle, respectively. The metallic palladium phase has been identified from the diffraction peaks of (111), (200), (220), (311) and (222) facets of cubic Pd at Bragg angle values of 40.4°, 47.0°, 68.5°, 82.0° and 86.4°, respectively (Fig. 1), which correspond well with the standard pattern (BD-01-088-2335). This observation confirms the presence of metallic Pd with an *fcc* structure. The diffraction peaks emerged at 2θ values of 36.7°, 43.0°, 62.4° and 75.0° corresponding to the reflection planes (111), (200), (220) and (311) respectively, thereby indicating the formation of pure CoO in the cubic crystal structure, with space group Fm3̅m as analyzed with ICSD database card no. 53057. All XRD patterns are dominated by the crystalline peak at 2*θ* = 36.7° from Pd suggesting the high crystallinity of the palladium phase. These XRD patterns do not indicate any peak from any other phase of cobalt oxide (Co_3_O_4_) or palladium, which indicates the purity of the deposit even on the prolonged deposition times of 3 h.

**Figure 2:**
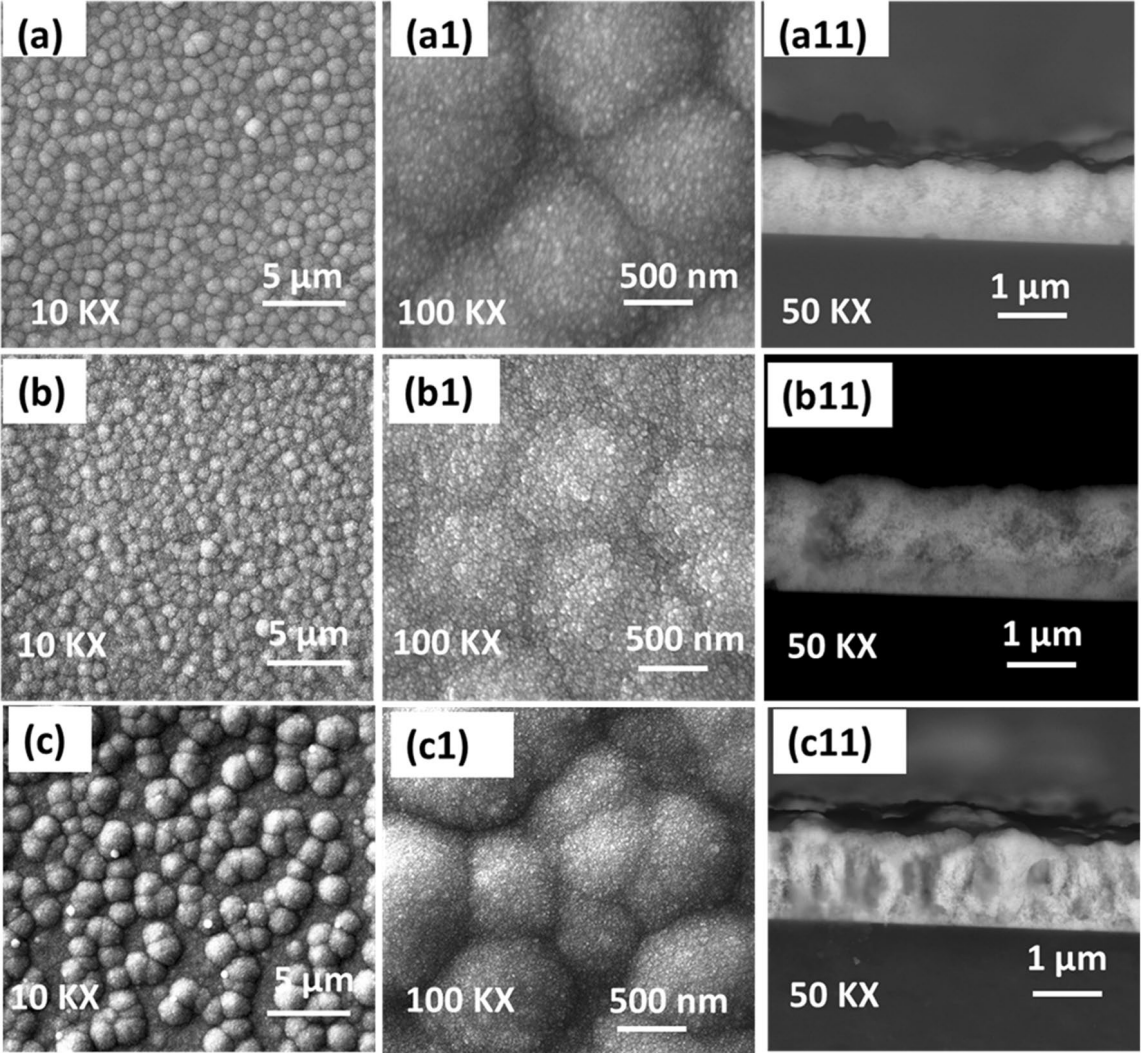
Surface and cross-sectional FE-SEM images of granular Pd–CoO films deposited on the plain glass substrates at temperatures of 475 ˚C for different deposition times of (**a**) 1-h (**b**) 2-h and (**c**) 3-h via. AACVD.

Figure 2 illustrates the surface and cross-sectional FE-SEM images of composite thin films formed at differing deposition times. The low resolution images 2 (a), (b), and (c) were taken in order to observe the surface coverage, the overall surface character, and the growth patterns. For all three films, the surface coverage is complete and homogenous. Moreover, all the surfaces are composed of evenly distributed spheres with well-defined grain boundaries. The diameter of these spheres in the case of PdCo-1hr and PdCo-2hr is in the range of 500 nm–1 µm; however, it has almost doubled in the case of PdCo-3hr, which is the result of coalescing phenomena very common in the film fabrication using AACVD. In fact, this coalescing effect is also visible in the case of PdCo-2hr, where the grain boundaries have started to merge or diminish. The corresponding high-resolution images (a1), (b1) and (c1) reveal that these microspheres are actually the granules composed of hundreds of small nanoparticles again with clear grain boundaries. Interestingly, the size of these tiny nanoparticles doesn’t change even when the granules are coalescing together from PdCo-1hr to PdCo-3hr, although the packing inside the granules becomes more and more compact. The cross-sectional images were also collected, which can depict the growth patterns and overall texture of the films. The film formed in the first hour of deposition is ~ 1 μm in thickness with a spongy appearance. (Fig. 2a11). PdCo-2hr also showed a similar appearance while growing to a size of ~ 1.5 μm but with some hardened spots in between, as shown in Fig. 2b11. However, after coalescing together, the PdCo-3hr was observed to be fully converted into a compact structure (Fig. 2c11), giving it a rocky texture while the size remains to be ~ 1.5 μm.

Elemental stoichiometry of the Pd–CoO composite was determined by conducting EDX analysis over a number of small and large areas of the films. Figure S1 indicates the recorded EDX spectra, which estimates the elemental stoichiometry of Pd:Co as ~ 1:1, which is in accordance with our synthetic strategy. Further, the presence of both cobalt and palladium elements in the films was ascertained by conducting EDX mapping. Figure S2 indicates the uniform distribution of Co and Pd elements in all of the films. Such homogenous distribution of the two elements, even in small 4–5 nm-sized nanoparticles, is extremely important for the bimetallic synergy to contribute to the water oxidation kinetics. The catalytic reaction occurs right at the interface, and a homogenous distribution provides the maximum number of those active interface sites. Therefore, the prepared films were expected to perform efficiently in different catalytic reactions and were further tested for the critically important OER in water splitting.

**Figure 3:**
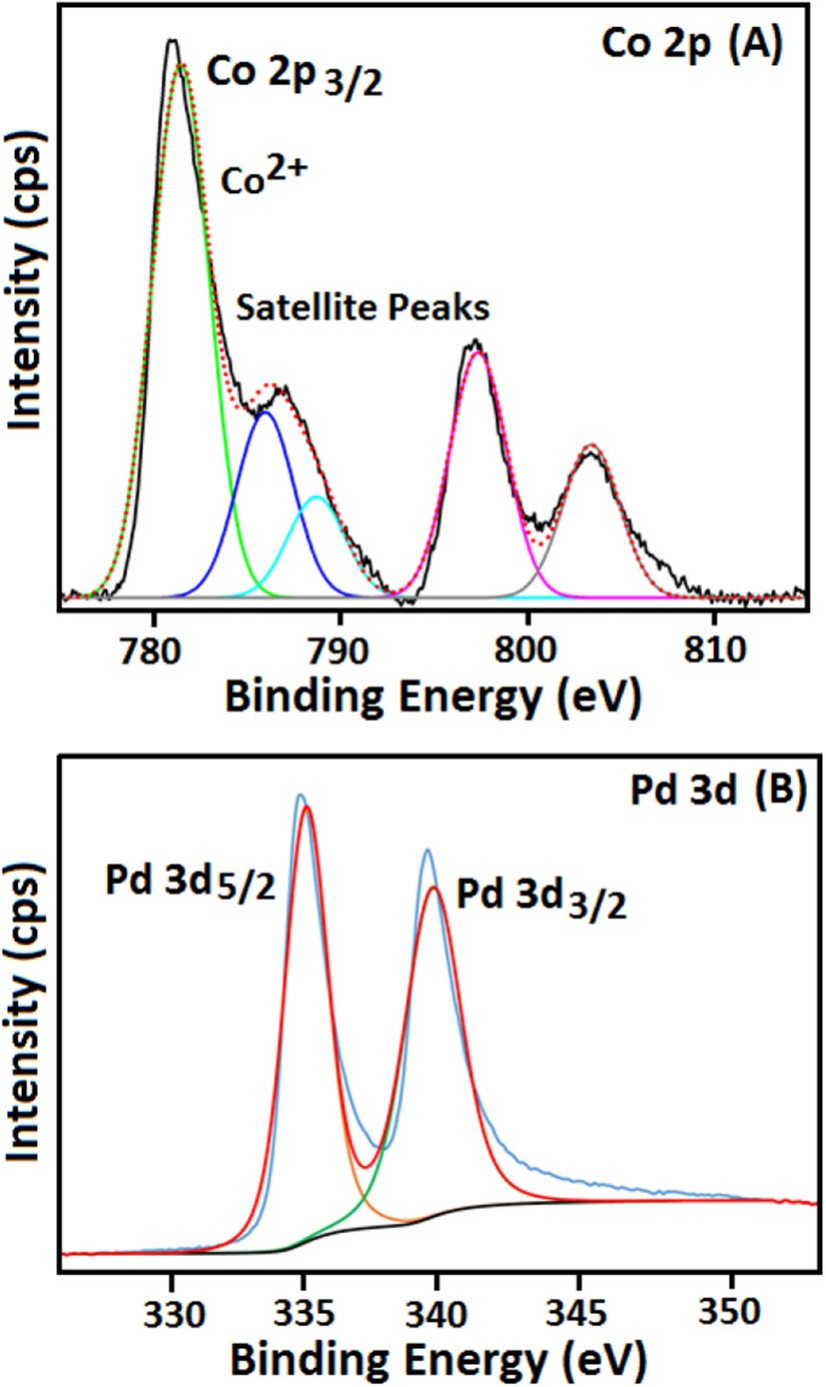
High-resolution XPS spectra showing the oxidation states of the constituents elements involved in Pd–CoO films indicating (**A**) Co 2*p* with oxidation state Co^2+^; (**B**) Pd 3*d* in (0) oxidation state.

Further, the oxidation states of constituent elements in the prepared Pd–Co composite film was studied by the high-resolution scan of XPS analysis. The high-resolution deconvoluted spectra of these individual elements are provided in Fig. 3. The binding energy 781.1 eV of Co 2*p* spectra can be fitted to Co^+2^ (Fig. 3A). Moreover, two satellite peaks are indicated at binding energies of 786 and 788 eV, which are characteristic of high-spin Co^+2^^24^. The doublet shown in Fig. 3B at the binding energy values of 333.5 eV and 339.0 eV is depictive of Pd 3*d*_5/2_ and Pd 3*d*_3/2_, respectively. These binging energy values are in agreement with the literature for metallic palladium^39,40^. In some studies, minor shoulder peaks at higher binding energies have also been shown, which are indicative of oxide formation on the Pd surface. However, the absence of these shoulder peaks in here is a confirmation of the absence of palladium oxide in the film. Furthermore, the unsymmetrical peak of O 1*s* can be indicated as in Fig. 3C. The high-resolution signals at a binding energy of 531.5 eV in the O 1*s* spectrum are shown as a characteristic feature for O that is bonded to the metal in metal oxide form. All these high-resolution spectra confirm the formation of a composite film of Pd and CoO in a very homogenous distribution.

### Electrochemical water oxidation studies

**Figure 4:**
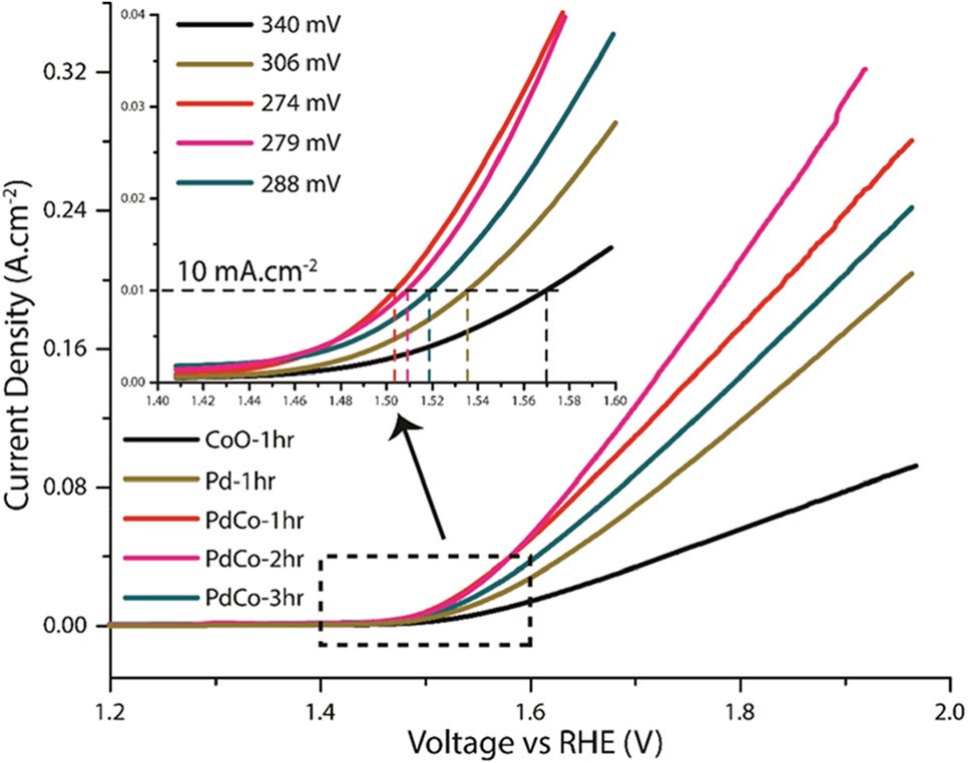
LSV curves for OER studies using different bimetallic Pd–Co, pristine CoO, and pristine Pd films fabricated on Ni-foam substrates in 0.1 M KOH. Inset is a zoomed part of the figure showing a quick rise of the current density beyond the onset potential of OER, also indicating the overpotential values for achieving a standard current density of 10 mA cm^−2^.

The completely characterized Pd–CoO films were re-deposited on Ni foam under similar AACVD conditions for the evaluation of OER activities, and a series of electrochemical measurements were performed. Figure 4 shows the LSVs recorded under a forward biasing (E) for three different films of bimetallic composites as well as their pristine components (CoO and Pd) prepared via AACVD for the sake of comparison in their OER activities. These experiments were performed in 0.1 M KOH solution at a scan rate of 5 mV s^−1^, and all the potentials were converted to the potentials vs. RHE for convenient comparison. The material loadings were also determined which comes out to be 0.41 mg cm^−2^, 0.37 mg cm^−2^, 0.43 mg cm^−2^, 0.52 mg cm^−2^, and 0.73 mg cm^−2^ for CoO, Pd, PdCo-1hr, PdCo-2hr, PdCo-3hr respectively.

As can be seen from Fig. 4, the fabricated bimetallic films, all three of them show better catalytic performance than their pristine counterparts, even when one of those is a highly active noble metal. The onset potential of all the films was lesser than 1.5 V vs. RHE, which is considered remarkable. This onset potential moves towards more negative values while we move from pristine CoO to pure Pd and then the composite films in the following order: PdCo-3hr > PdCo-2hr > PdCo-1hr. However, the more important feature of the bimetallic films was the higher current density as compared to Pd and CoO which quickly rises in a small potential window and reaches to the value of more than 300 mA cm^−2^ in the case of PdCo-2hr, while the potential is still under 1.9 V vs. RHE.

The inset from Fig. 4 shows a zoomed-in part of the LSV curves to better indicate the potentials required to achieve a current density of 10 mA cm^−2^. A sustained current density equal to this value is a pre-requisite for at least a 10% conversion of electric energy into chemical fuels. The inset also enlists the overvoltage values to achieve this target current density. PdCo-1hr shows the lowest over-potential of 274 mV as compared to PdCo-2hr = 279 mV, PdCo-3hr = 288 mV, Pd = 306 mV and CoO = 340 mV. This over-potential is much lower than the benchmark value of 350 mV and is better than many proposed materials based on Co and Pd listed in Table 1 with low Pd-loading, as in this case. This high performance of the bimetallic film, especially the one prepared in 1 h and 2 h of deposition, can only be attributed to the two factors. First is the uniform distribution of Pd and Co in the synthesized films in a porous and spongy appearance, allowing better access to the reacting species for the catalytic sites (From SEM image Fig. 2a11). The current densities become a little higher for higher metal deposition in PdCo-2hr corresponding to greater metal loadings, yet, these effects are not significantly larger to attract greater attention. A decrease in the performance is observed in the PdCo-3hr film can be related to this factor, as the film has become solid and dense after the enhanced sintering time. Moreover, this depicts that the active layer with sufficient reaction centers linked to the Pd electron sink has already been deposited after about 1–2 h of AACVD operation. Second, and more important is the synergy of the two metals, one in the pure form and other in the oxide form that can lead to even better performance than the pure Pd film itself. In our recent publications^24,41^, we have discussed a plausible mechanism of the catalytic process with Co-based materials, which involves an activation of Co sites in the alkaline media. The readily oxidizable Co(II) is converted to Co(III) and then transit into the Co(III)-OH form, which could catalyze the oxidation with OH^−^ species acting as active sites. Further oxidation of these active species lead to the formation of Co(IV) linked oxygen complex. Another transition of the adsorbing OH^−^ species forms the Co(IV) linked OOH complex. This OOH complex reacts with OH^−^ again to generate oxygen and is returned to the Co(III)-OH state. We have shown these phenomena in both pure CoO materials and Co-composites with other metals. In this work, the catalytic pathway of Co is changed in the presence of Pd, which benefits the catalytic activity of the proposed material during the OER. This has recently been proposed that the metallic Pd allow strong adsorption of oxidation targets, which in combination with the Co-affinity of oxygen, drives the surface composition changes highly valuable for the catalytic processes^20^. The oxygen atoms which are part of CoO can migrate to react with the oxidizable molecules on the neighboring Pd atoms to form catalytic products. O atoms on the Pd-supported CoO layer can also react with these oxidizable molecules, producing O-vacancies while Pd acting as an electron sink. This results into further activation of Co for another adsorption and reaction event. However, both these phenomena only occur at a sufficiently high loading of Pd with only a monolayer of Co which corresponds to Co_0.24_Pd_0.76_ shown in this reported work^23^. Though this content ratio can be brought to ~ 1:1 (i.e., Co_0.46_Pd_0.54_ for PdCo-2hr), which has been shown here for water oxidation, and still it displays to be a highly active catalyst. Such a catalyst with low precious metal loading is highly beneficial in decreasing the cost of catalytic materials for different applications. This is truly remarkable when the rapid time frame of the deposition is considered, as it only takes between 60–120 min to fabricate extremely efficient bimetallic films without the need for any further immobilization or any prior template-based manipulations.

**Table 1:** Comparison of the electrocatalytic water oxidation performance in alkaline conditions using CoO based materials either in pristine form or in combination with noble metals.

Catalyst	Electrode	Overpotential (10 mA cm^−2^)	Overpotential (100 mA cm^−2^)	References
CoO hexagrams	GC	269 mV	410 mV	^45^
CoO NP	Ni foam	497 mV	646 mV	^46^
CoO NS	Carbon cloth	320 mV	395 mV	^47^
PGE-CoO	GC	348 mV	460 mV	^48^
CoO NS @p-Si	p-Si substrate	560 mV	> 770 mV	^43^
Single Crystal CoO NR	Carbon fiber paper	330 mV	> 470 mV	^42^
IrO_2_	GC	330 mV	Not reported	^49^
NiCoO_2_ (rock salt)	GC	390 mV	Not reported	^10^
RuO_2_ (rutile)	GC	380 mV	Not reported	^10^
Pd–CoO granules	Ni Foam	274 mV	430 mV	This work

**Figure 5:**
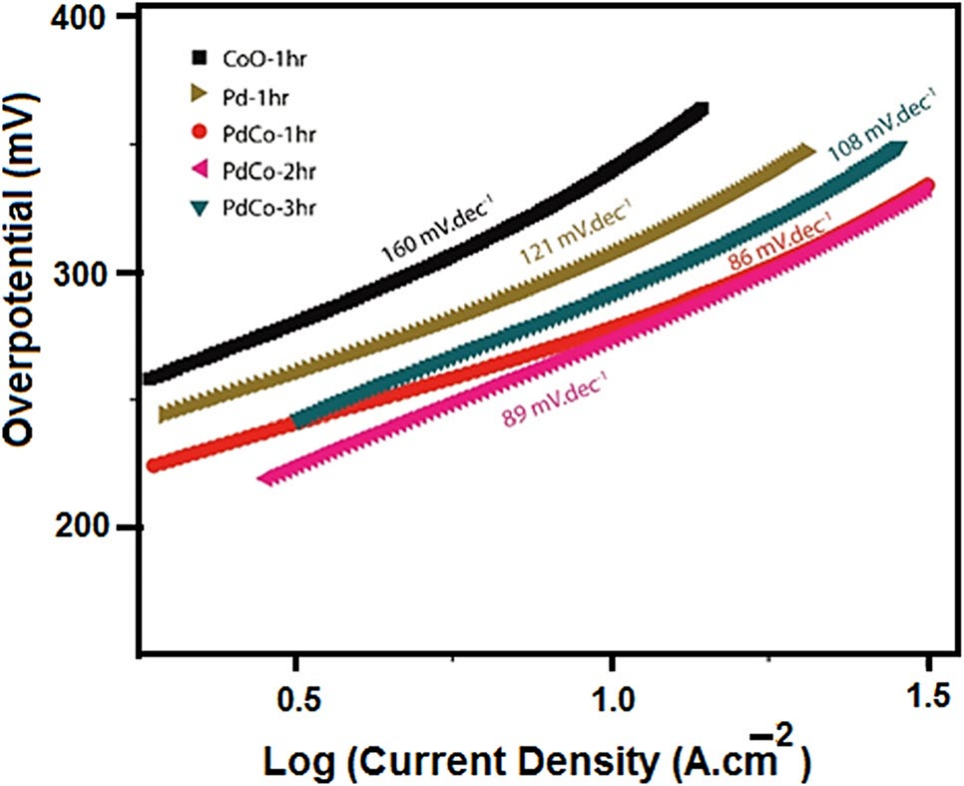
Taefl plots for as-synthesized pristine CoO, pristine Pd and bimetallic PdCo-1hr, PdCo-2hr, and PdCo-3hr showing the Taefl slope values in mv dec^−1^.

The current densities were also compared at 100 mA cm^−2^ and 200 mA cm^−2^ as shown in Fig. S3. 100 mA cm^−2^ current density was attained by all three bimetallic films and the Pd film easily below a potential of 1.8 V vs. RHE; however, the PdCo-2hr film shows the best performance here achieving this target at 430 mV. 200 mA cm^−2^ of current density, on the other hand, was achieved only by PdCo-2hr film while still under the potential of 1.8 V vs. RHE, and the overpotential, in this case, was 550 mV. This actually satisfies one of the requirements for commercial or industrial scale materials that need to generate 200–400 mA cm^−2^ current density while the applied potential is between 1.8–2.4 V vs. RHE^2^ Many robust electrocatalyts to be used in alkaline electrolyzers have failed to attain this performance level. This performance is particularly limited in case of reported CoO materials^42,43^, rarely indicating the achievement of current density > 200 mA cm^−2^. The high efficiency of proposed PdCo materials is further depicted by drawing the Tafel plots, which can show the rate of water oxidation reaction in the given range of potential. The resulting Tafel plots for all the PdCo films within the same region of electrochemical scanning at a rate of 5 mV s^−1^ are shown in Fig. 5 alongside the data of fitting Tafel equation indicating the sustainability of the reaction rates. A small Tafel slope is an expression of well-balanced kinetics during catalysis^44^ which was the case for the PdCo-1hr and PdCo-2hr bimetallic films, differing only slightly from each other. This demonstrates sufficiently high kinetics for both of those films. These slope values were much lower than individual Pd or CoO films, which corresponds to the lower performance of single component films as compared to bimetallic synergistic films. However, the working range of the PdCo-1hr film is wider while still having the lowest Tafel slope of 86 mV dec^−1^. This depicts the relation of the catalytic performance to the active sites in the surface structure of the material. This film being least dense from the lot, shows the highest current densities with lower Taefl slope. An open structure and bimetallic synergy support the fast mass transport and boost the electron transfer without undergoing any scattering losses, as higher number of accessible catalytic sites are readily available.

Measurement of the active surface area can be regarded as an important factor in catalytic materials which provide an approximation of the available catalytic sites. This factor was numerically assessed by the double-layer capacitance measurements of the film. Charging current densities within a non-faradaic potential region were plotted against the scan rates (Fig. S5), and the slopes were divided with the geometric area of the electrode to get estimated ECSA values. A significantly higher linear slope value of PdCo-1hr and PdCo-2hr films (i.e., 3.67 and 4.49 mF respectively) as compared to all other films fabricated in this work indicates that these two films have the highest number of active sites available, thereby making the catalytic reaction kinetically favorable. All these performance parameters were also compared with literature values where oxides of cobalt or their combination with Pd is used as water oxidation catalyst (Table 1), and it was found that this is the first-ever example of using Pd–CoO combination in the water electro-oxidation and also the first example of such kind of material produced via AACVD, a simple, rapid, and efficient protocol for material synthesis.

When the PdCo-2hr film is subjected to a scan rate study (shown in Fig. S4), it is found that the increase of scan rate from 1 to 100 mV s^−1^ has no effect on the onset potential as well as the overpotentials to achieve different current densities. For all different scan rates, the current density of more than 200 mA cm^−2^ is achievable below 1.8 V vs. RHE. The only difference that is visible for measurements at different scan rates is the shoulder peak that arises from the conversion of M^+2^ to M^+3^ and is reported in many studies based on Ni foam. The anodic current due to this oxidation peak rises with the scan rate and make it harder to estimate the overpotential for lower current densities. Therefore, 5 mV s^−1^ scan rate is selected for all the calculations where this shoulder peak is sufficiently small. It was also observed at lower scan rates, where this anodic peak is absent, the onset potential, as well as the overpotential, is slightly negative as compared to the higher scan rates. This means that the slower kinetics of the reaction allows more time for the target species to access the catalytic sites and to form the oxidized products.

**Figure 6:**
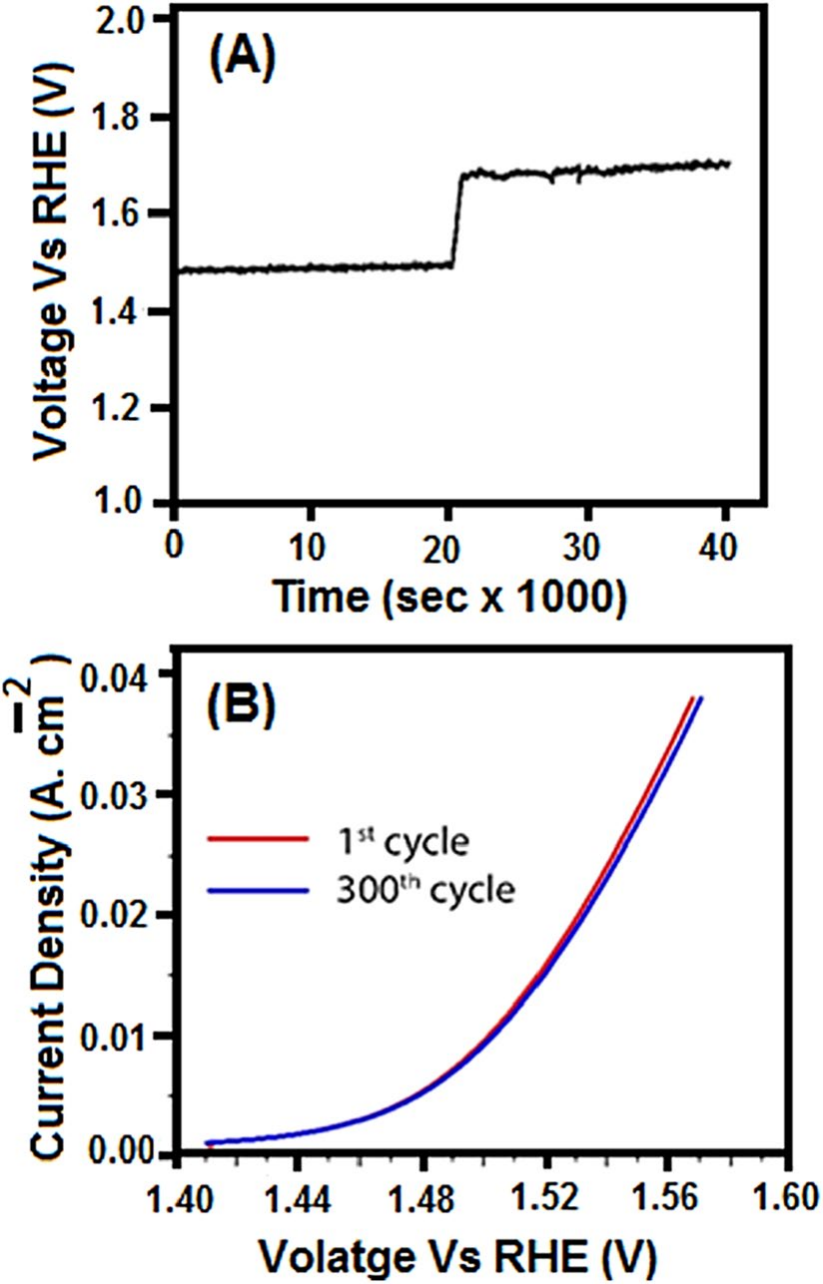
Stability tests of PdCo-1hr film fabricated via AACVD where (**A**) depicts a 40,000 s experiment for a couple of fixed current density values to obtain corresponding voltages thereby obtaining a constant signal and (**B**) depicts a set of LSV experiments in multiple cycles and the data shown is for the first cycle and 300th cycle while still overlapping.

Apart from the excellent OER performance, the PdCo-1hr film demonstrated long term stability and durability, which are essential to evaluate the performance of an electrocatalyst. Figure 6A shows the long-term performance of this catalytic film for OER in 40,000 s to generate constant current densities at two different levels. Current density values of 25 mA cm^−2^ and 80 mA cm^−2^ were identified, and the voltage signals to obtain these current densities were recorded. It was observed that the voltage signals remained stable at the values of about 1.55 V and 1.65 V, respectively, proving a constant rate of production of oxygen via catalytic water splitting. This rate of retention for the current is far better than reported RuO_2_ materials. Furthermore, signal stability was also determined after multi-cycle measurements. Figure 6B indicates a comparison of the 300th cycle to the 1st, and the signals are highly overlapping.

Another component of the stable character of the catalytic materials is to observe and understand the structural and morphological changes caused by long term electrochemical cycling. In order to perform this, the PdCo-1hr film was subjected to SEM, XRD, XPS, and elemental analysis after 300 cycles of measurement. Figure S6 shows the topological surface characters of this film, indicating that the granules of the material remained intact with visible grain boundaries, although, a bit distorted as compared to the original structure (Fig. 2a). The particle size of the composite material did not change as well, which is an indication of the overall stability of the films furnished from their long term experiments. Elemental mapping and EDX analysis shown in Fig. S7 also shows that both the constituent elements of the composite in a well-distributed form are present. However, the percentage of Co in the film has deviated to a lower value from 1:1 ratio, which is the result of the inherently less stable nature of the cobalt oxide phase. This means that either the CoO phase has undergone oxy or hydroxyl transformation under the influence of electrochemical oxidation reaction and/or leaching of the Co has occurred. Therefore, XPS analysis of the PdCo-1hr electrode was recorded to predict the oxidation states of the constituent elements, as shown in Fig. S8. The XPS spectrum of the Pd has not changed significantly, which is due to the inherently stable and noble nature of this metal. The de-convoluted peaks of Co 2P spectra, though, indicate that both Co^+2^ and Co^+3^ oxidation states are present in addition to Co(OH)_2,_ which depicts that the Co(II) oxide is going thorough different transformations as a function of reaction cycles. However, this all has not significantly reduced the catalytic activity even after 300 cycles characterizing the overall stability of the material. Such extraordinary stability, when added to high efficiency, makes these catalytic materials a favorable choice in commercial applications. The ease of preparation via AACVD is also proven to be a smart and highly efficient method to design exotic and characteristic nanomaterials and films, and many such fabrication processes are underway in our lab.

## Conclusions

In summary, the bimetallic PdCo granular films composed of tiny nanoparticles having exquisite 1:1 distribution of both metals have been successfully fabricated through a single-step AACVD procedure. It was found that the texture and morphology of the final product can be controlled by the deposition time. The as-synthesized Pd–Co films exhibited high electrocatalytic activity and favorable stability toward oxygen evolution reactions in water splitting. In this way, an efficient approach was established for the controlled design and synthesis of synergistic bimetallic films. The correlation between catalytic performance and the material texture indicates that coexisting Pd and CoO provide the active sites which are more readily available in the case of a spongy film as compared to a rocky one. As a result, only 1–2 h of fabrication process lead to highly efficient catalytic materials promoting water oxidation kinetics to a level of commercial standards. By utilizing the same strategy, various bimetallic and trimetallic materials can be further synthesized in a controllable manner to be applicable in various catalytic and sensing studies.

## Supplementary information

Supplementary Information.
