# Supplementary material for: Synergistic effects in bimetallic Pd–CoO electrocatalytic thin films for oxygen evolution reaction
Source: Sci Rep. 2020 Sep 2;10:14469. doi: 10.1038/s41598-020-71389-w (PMC7467929; doi:10.1038/s41598-020-71389-w)
Supplement: Supplementary file 1 — Supplementary Information. [file 41598_2020_71389_MOESM1_ESM.docx]

**Synergistic Effects in Bimetallic Pd-CoO Electrocatalytic Thin Films for Oxygen Evolution Reaction**

Muhammad Ali Ehsan,^1^ Abbas Saeed Hakeem,^1^ Abdul Rehman ^2,^*

[1] Center of Research Excellence in Nanotechnology (CENT), King Fahd University of Petroleum & Minerals, Dhahran 31261, Saudi Arabia.

[2] Department of Chemistry, King Fahd University of Petroleum and Minerals, Dhahran 31261, Saudi Arabia

* Corresponding Author, Email: abrehman@kfupm.edu.sa

**Supplementary Information**


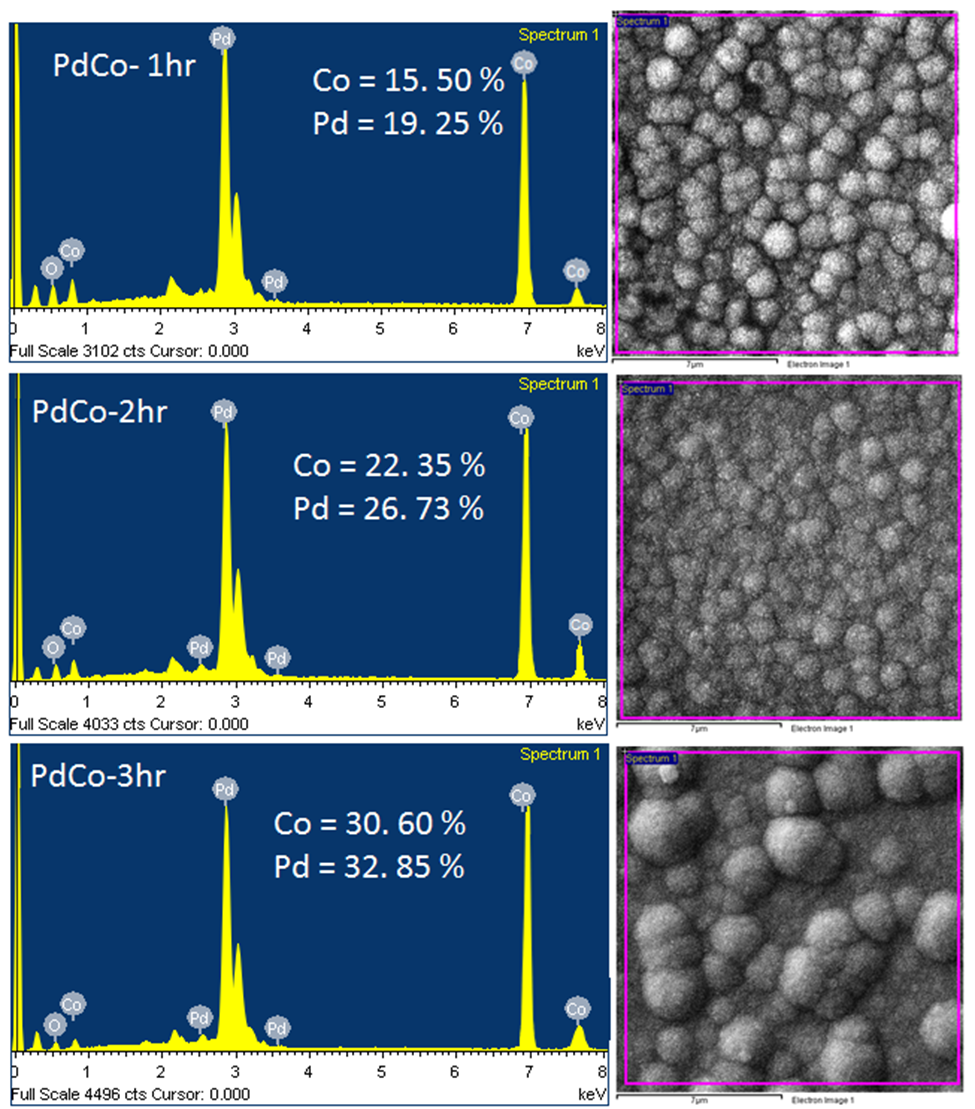


**Fig. S1:** EDX spectra of Pd-CoO composite films prepared in different deposition time durations of 1hr, 2hr and 3hr with higher-resolution images obtained via SEM.


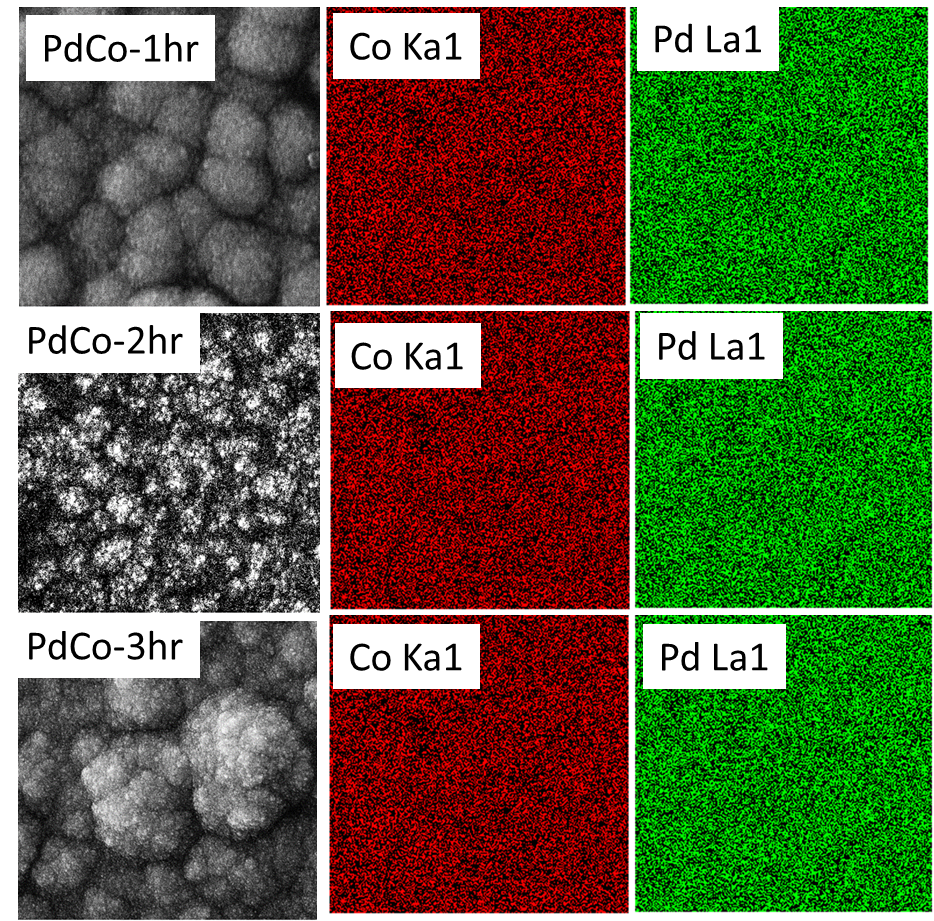


**Fig. S2:** EDX elemental map showing the homogenous distribution of Co and Pd atoms in Pd-CoO composite films made 1hr, 2hrs and 3hrs.


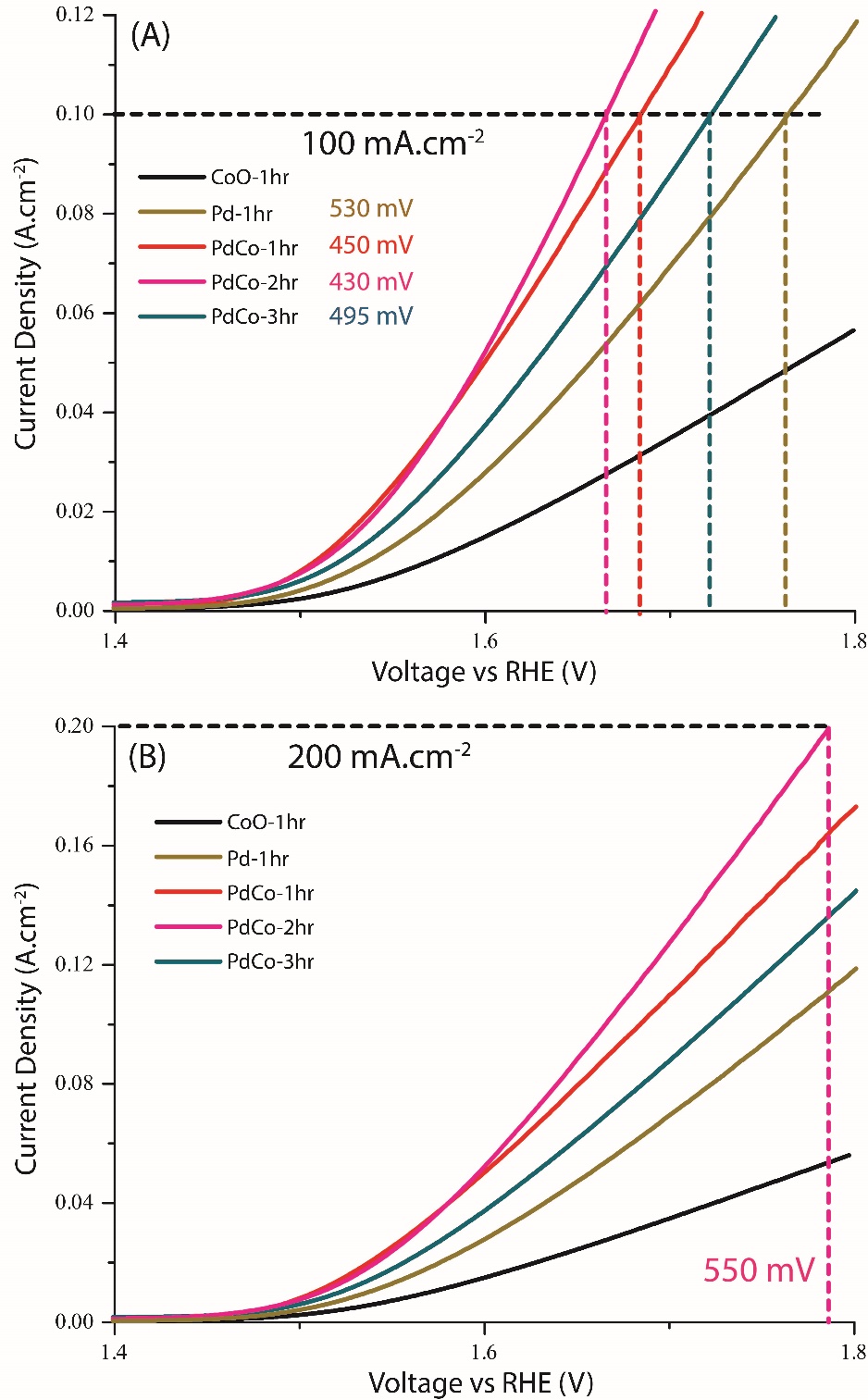


**Fig. S3:** LSV curves for different films prepared via AACVD showing the attaining of higher current densities of (**A**) 100 mA.cm^-2^ and (**B**) 200 mA.cm^-2^. 100 mA.cm^-2^ current density was attained by all three bimetallic films and the Pd film easily below a potential of 1.8 V vs RHE, however, the PdCo-2hr film shows the best performance here achieving this target 430 mV. 200 mA.cm^-2^ current density on the other hand was achieved by only PdCo-2hr film while still under the potential of 1.8 V vs RHE and the overpotential in this case was 550 mV.


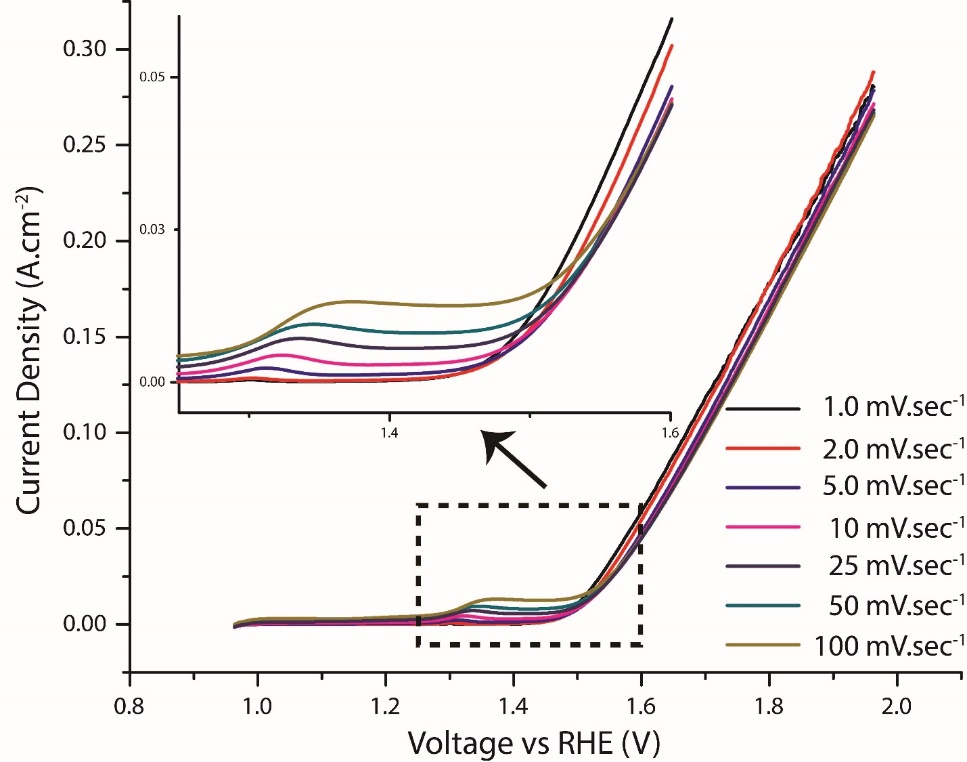


**Fig. S4:** LSV curves for PdCo-2hr film at different scan rates whereas the inset is showing a zoomed in part of those curves.


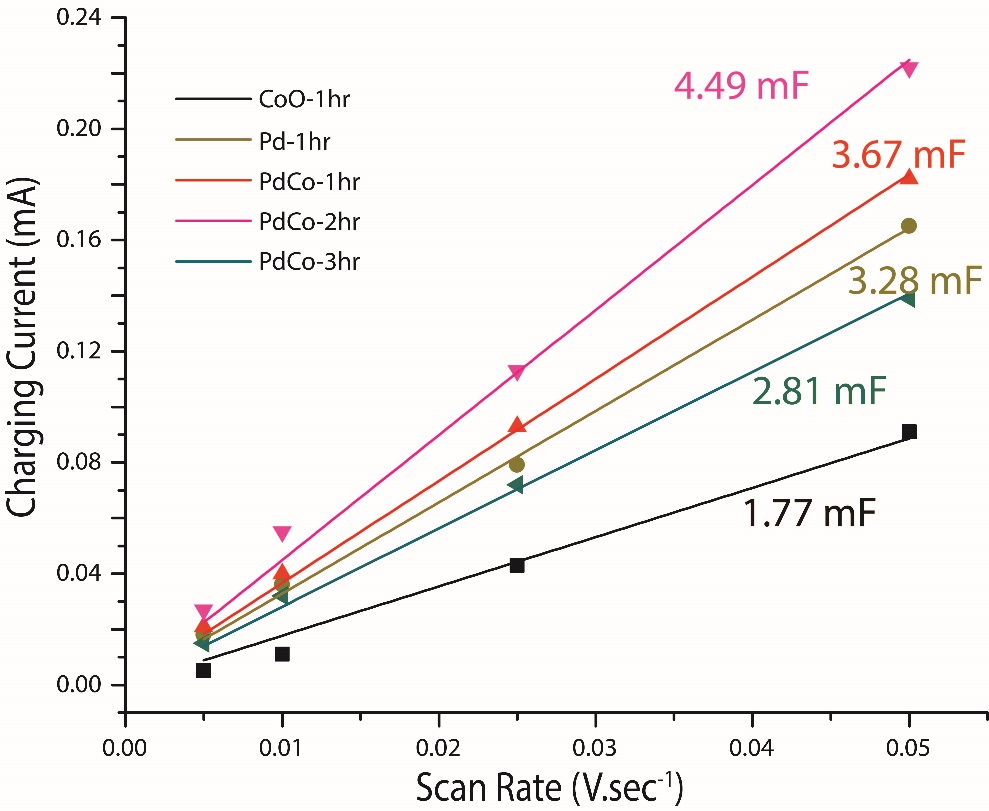


**Fig. S5:** The charging current vs the scan rate plots for experiments in the non-faradaic zone and the calculated slope values for different films of the catalytic materials prepared in this work.


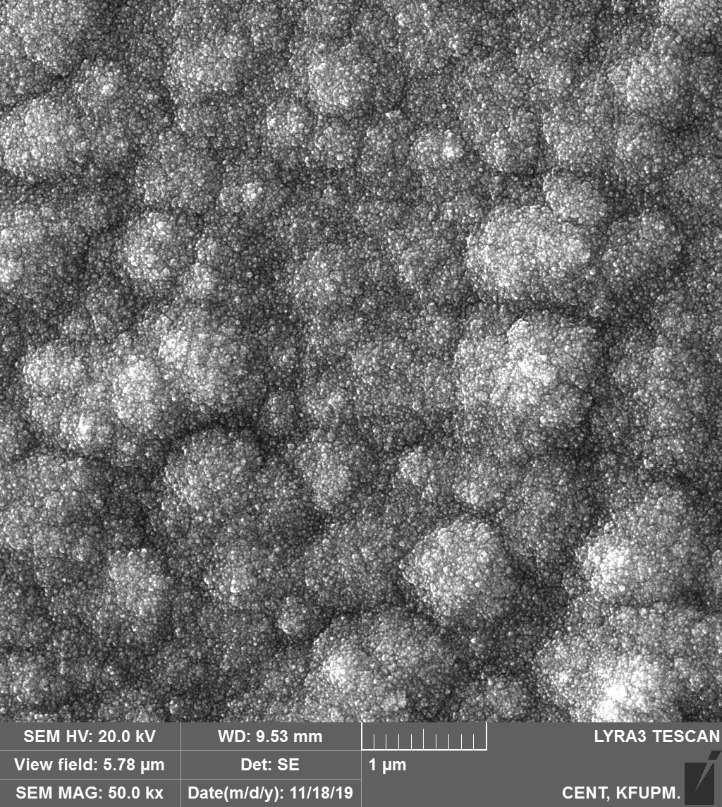


Fig S6: SEM image of PdCo-1h film after prolonged electrochemical stability tests.


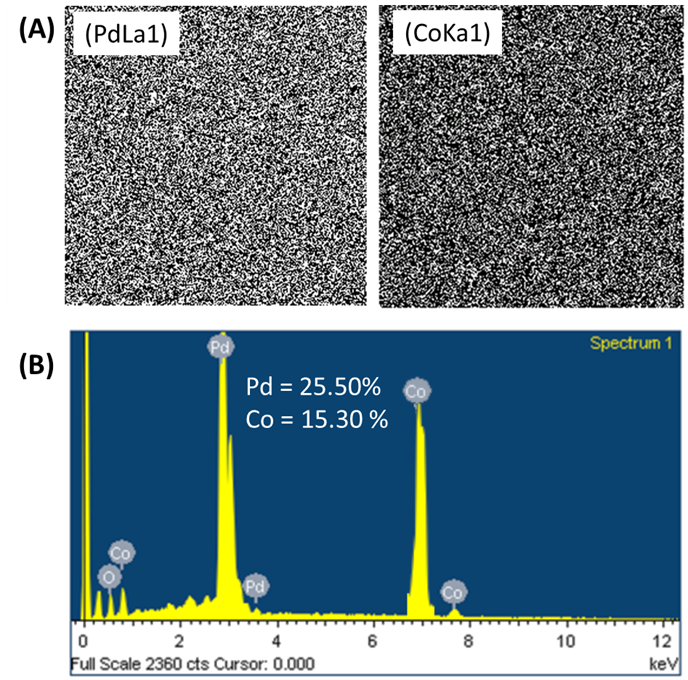


Fig S7: (A) Elemental map of PdCo-1hr catalyst after the stability experiments showing the presence of Pd and Co elements in the film; and (B) EDX spectrum of the same film.


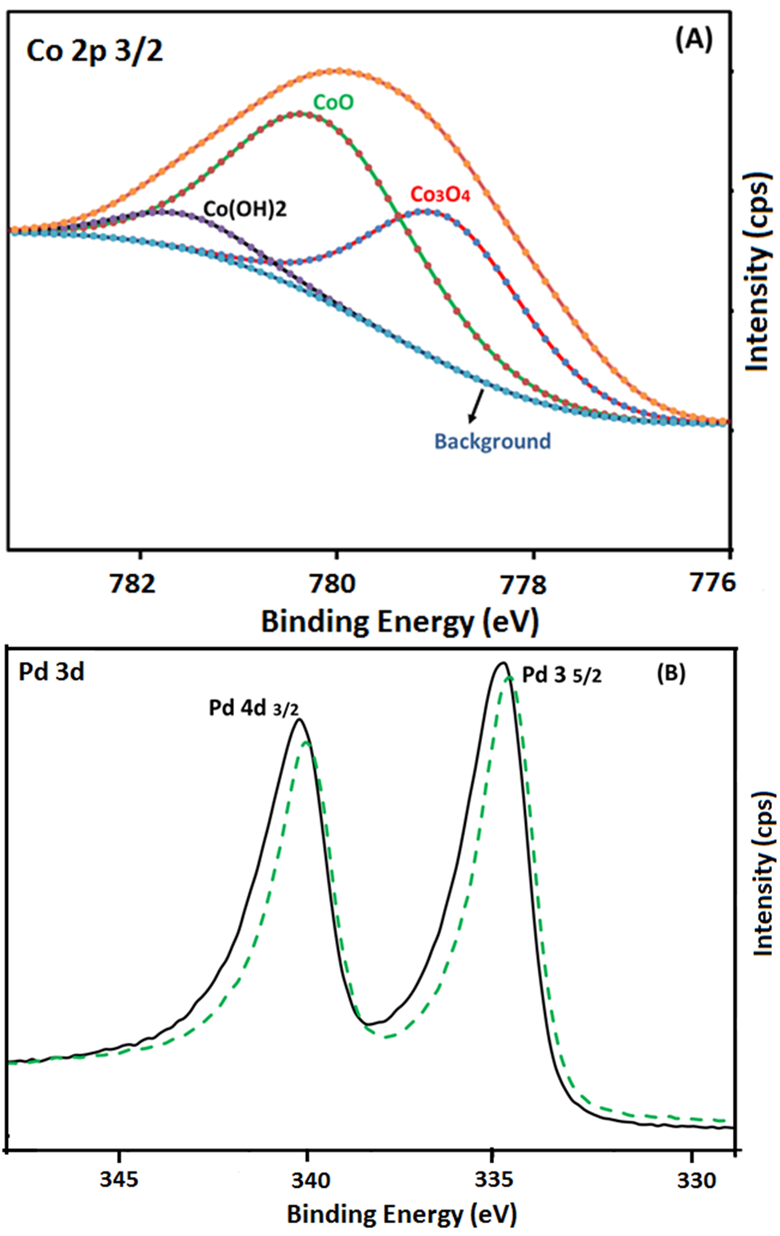


Fig S8: High resolution XPS spectra of showing the oxidation states of the constituents elements involved in PdCoO-1hr after stability test (A) Co 2p with oxidation state Co^2+^ and Co^3+^ (B) Pd 3d in (0) oxidation state.
